# Insulin Resistance and Skin Diseases

**DOI:** 10.1155/2015/479354

**Published:** 2015-04-21

**Authors:** Maddalena Napolitano, Matteo Megna, Giuseppe Monfrecola

**Affiliations:** Section of Dermatology, Department of Medicina Clinica e Chirurgia, University Federico II, Napoli, Italy

## Abstract

In medical practice, almost every clinician may encounter patients with skin disease. However, it is not always easy for physicians of all specialties to face the daily task of determining the nature and clinical implication of dermatologic manifestations. Are they confined to the skin, representing a pure dermatologic event? Or are they also markers of internal conditions relating to the patient's overall health? In this review, we will discuss the principal cutaneous conditions which have been linked to metabolic alterations. Particularly, since insulin has an important role in homeostasis and physiology of the skin, we will focus on the relationships between insulin resistance (IR) and skin diseases, analyzing strongly IR-associated conditions such as acanthosis nigricans, acne, and psoriasis, without neglecting emerging and potential scenarios as the ones represented by hidradenitis suppurativa, androgenetic alopecia, and hirsutism.

## 1. Introduction

The skin is the major border organ of human body, being the most exposed to environmental variations. However, it also offers a window to what is going on inside the body so that changes to the skin may signal a more serious health problem, frequently serving as a marker for underlying internal disease [1, 2]. Numerous internal diseases are able to present cutaneous manifestations which may precede, occur concurrently with, or follow the onset of the internal conditions. There are a huge number of studies regarding the relationship of the most common skin manifestations of internal diseases (e.g., diabetes, inflammatory bowel diseases, lupus erythematosus, systemic sclerosis, and tumors) [1, 2]. However, the surveys regarding the relationship between metabolic alterations such as insulin resistance (IR) and dermatologic conditions are still scant.

In this review, we will discuss the principal skin diseases and dermatological conditions which have been linked to IR, analyzing the mechanisms of the connections between cutaneous and metabolic deregulations (Table 1).

## 2. Insulin, Insulin Resistance and Skin

**Figure 1:**
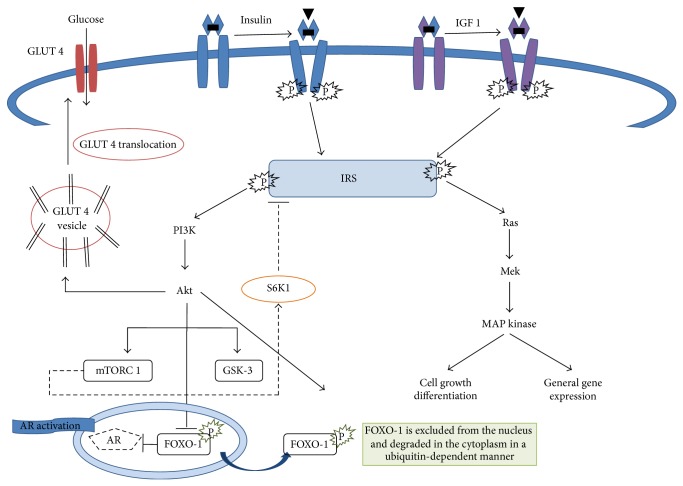
Insulin signalling pathway: insulin binds to the insulin receptor leading to its autophosphorylation and recruitment of adaptor molecules such as insulin receptor substrates (IRS 1–6) to engage multiple downstream signalling pathways. IRS activation can be also triggered by IGF-1 signalling. Phosphoinositide 3-kinase (PI3-K)/Akt, mammalian target of rapamycin (mTOR), and the Ras/mitogen-activated protein kinase (MAPK) pathways represent the major cellular signalling pathways activated. Particularly, AKT controls the assembly of glucose transporter- (GLUT-) 4 at the cell membrane and thus controls glucose influx into the cell. mTOR complex 1 (mTORC1) activates the kinase S6K1, which phosphorylates and inhibits IRS and thus reduces AKT-GLUT-dependent glucose uptake, the principal mechanism of peripheral insulin resistance. AKT phosphorylation pathway inhibits FOXO-1 mediated gene expression by its extrusion from the nucleus to the cytoplasm, preventing FOXO-1 mediated repression of androgen receptors. GSK-3 = glycogen synthase kinase 3 and FOXO-1 = forkhead box protein O-1.

**Figure 2:**
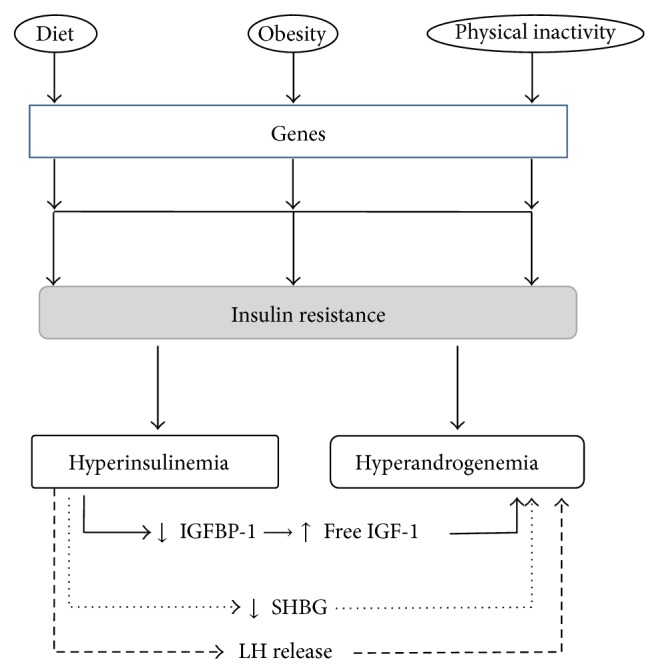
Connections between insulin resistance, hyperinsulinemia, and hyperandrogenism. FSH = follicle stimulating hormone, IGF = insulin-like growth factor, IGFBP = insulin-like growth factor binding protein, LH = luteinizing hormone, and SHBG = sex hormone binding globulin.

Insulin, a polypeptide hormone produced by the beta cells of the islets of Langerhans of the pancreas, controls the level of the glucose in the blood so is a central player in the metabolic system. Insulin binding to the insulin receptor leads to receptor autophosphorylation and recruitment of adaptor molecules such as* insulin receptor substrates* (IRS 1–6) or Shc which are in turn phosphorylated and serve as binding sites to initiate the activation of different signaling cascades including the mitogen-activated protein kinase (MAPK) and phosphoinositide 3-kinase (PI3-K) pathways [3]. These pathways not only regulate glucose, lipid, and protein metabolism, but also control mitogenic responses through the control of proliferation, differentiation, and apoptosis (Figure 1). Insulin signaling is downregulated through inhibitory serine phosphorylation of IRS 1, thus rendering the cells resistant to insulin. Interestingly, inflammatory mediators, such as cytokines, can induce IR through the activation of IRS kinases [4]. Furthermore, insulin has an important role in homeostasis and physiology of the skin, although the exact function of insulin signaling remains controversial. Under healthy conditions, insulin regulates the equilibrium between proliferation and differentiation of keratinocytes, a prerequisite for the formation of the epidermal structure. Under conditions of chronic inflammation (e.g., acne or psoriasis), high levels of proinflammatory cytokines activate p38MAPK, which induces IR by serine phosphorylation of IRS, leading to blockade of differentiation and, at the same time, to an increased proliferation of basal keratinocytes [3]. IR is defined clinically as the inability of a known quantity of exogenous or endogenous insulin to increase glucose uptake and utilization in an individual as much as it does in a normal population. It causes an insufficiency in insulin-stimulated glucose transport in the skeletal muscle and fat tissue, as well as a suppression of glucose production in the liver [5]. In addition, as a result of the IR, the pancreas produces much more insulin than normal. This condition, called hyperinsulinemia, accelerates lipogenesis with increased production of free fatty acids, reduces levels of sex hormone binding globulin (SHBG), increases luteinizing hormone (LH) and follicle stimulating hormone (FSH) levels, and, finally, leads to an increase in the production of ovarian androgens and also in their biologically active portion potentially leading to hyperandrogenism (Figure 2) [6, 7]. Hyperandrogenism, a common endocrine disorder of women of reproductive age with a prevalence of 5–10%, comprises a heterogeneous group of conditions that exhibit a common phenotype. The most frequent hyperandrogenic-linked disorder is the polycystic ovary syndrome (PCOS). It shows an 80–85% prevalence among women with excess androgen and is also closely linked to IR [8]. The clinical signs of hyperandrogenism are very important especially for the dermatologist since they include the following: hirsutism, alopecia, seborrhea, acne, and, in severe cases, signs of virilization (deepening of the voice, increased muscle mass, clitoromegaly, decreased breast size, and amenorrhea), highlighting the wide clinical scenario which is related to IR and hyperinsulinemia. As regards IR and hyperinsulinemia evaluation, although the glucose-insulin relationship is clinically relevant, it is also important to recognize that, theoretically, IR responds to influences other than glucose metabolism. The reference standard for the evaluation of insulin sensitivity is the glucose clamp test. However, this test is limited to research use and is difficult to perform at all medical institutions [9]. Homeostasis model assessment (HOMA), first described in 1985 by Matthews et al., is a method for estimating insulin sensitivity. It is calculated by multiplying fasting plasma insulin (FPI) by fasting plasma glucose (FPG), then dividing by the constant 22.5 [10]. Compared with the “gold” standard euglycemic clamp method for quantifying IR, quantification using HOMA is more convenient. This method has been applied across all ethnic groups. One study suggested that the range of normal HOMA-IR in a healthy Hispanic population may be higher than the one in Caucasians in central and north America, and certainly this population is known to have a genetic susceptibility to type 2 diabetes, which is closely associated with IR. Indeed, the best cutoff of HOMA-IR in Hispanic population seems to be 3.80 for the definition of IR. This is higher than the widely adopted cutoff of 2.60 for Caucasian population [11]. Therefore, in spite of its importance, the lack of a standardized reference range for HOMA-IR has hindered its clinical and population application. However, Katz et al. proposed a new formula to calculate insulin sensitivity that relies less on insulin levels, called the quantitative insulin sensitivity check index (QUICKI) [12]. Some authors have observed that QUICKI has a better correlation with the euglycemic clamp than HOMA-IR and a lower coefficient of variation. Sarafidis et al. and Antuna-Puente et al. reported a coefficient of variation for this index, based on two fasting glucose and insulin samples, of 7.8 and 3.9%, respectively [13, 14]. However, even considering these advantages, the formula is still rarely used in clinical studies compared to HOMA-IR.

## 3. Material and Methods

We searched for English-language literature describing the relationships between insulin resistance and skin diseases in the following commonly used websites: PubMed (http://www.pubmed.com/); Google (http://www.google.com/); Google scholar (http://scholar.google.com/); Scopus (http://www.scopus.com/); and EBSCO (http://www.ebsco.com/). The following keywords were used: insulin, insulin resistance, skin diseases, obesity, cutaneous diseases, diabetes, cutaneous manifestations, dermatologic conditions, internal diseases, and cutaneous disorders.

## 4. Skin Diseases Strongly Associated with IR

### 4.1. Acanthosis Nigricans

Acanthosis nigricans, a cutaneous condition affecting localized areas of the skin, is among the most common dermatologic manifestations of obesity and IR/hyperinsulinemia. Indeed, hyperinsulinemia is able to stimulate insulin-like growth factor (IGF) receptors with subsequent keratinocyte proliferation [15]. The activity of IGF-1 is regulated by IGF binding proteins (IGFBPs), which increase IGF-1 half life, deliver IGFs to target tissues, and regulate the levels of the metabolically active “free” IGF-1. IGFBP-1 and IGFBP-2 are both decreased in obese subjects with hyperinsulinemia, increasing plasma concentrations of free IGF-1. An increase in bioactive IGF-1 promotes cell growth and differentiation [16, 17]. IGF-1 is expressed within the stratum granulosum and by dermal fibroblasts, but not by epidermal basal keratinocytes. In theory, an insulin-induced systemic reduction of IGFBP-1 and IGFBP-2 could increase local levels of free IGF-1, thereby facilitating the development of hyperkeratosis and papillomatosis observed in acanthosis nigricans [18]. The prevalence of this condition varies from 7% to 74%, according to age, race, frequency of type, degree of obesity, and concomitant endocrinopathy. It is most common in Native Americans, followed by African Americans, Hispanics, and Caucasians [19]. This condition appears as symmetric, velvety, hyperpigmented plaques that may occur in almost any location. It is most commonly observed in the axilla, groin, and posterior neck but can also be seen on the elbows, knuckles, and face, particularly in ethnic skin. The hyperpigmentation observed is secondary to acanthosis and papillomatosis of the epidermis rather than pigment-producing cells [20]. Many classifications of AN have been proposed. Curth classified AN into benign (obesity related, hereditary, and endocrine forms) and malignant (associated with tumour) forms [21]. In 1994, Schwartz proposed a classification including benign and malignant forms, forms associated with obesity and drugs, acral acanthosis nigricans, unilateral acanthosis nigricans, and mixed and syndromic forms [22]. Burke et al. classified AN according to severity on a scale of 0–4 based on how many areas are affected. This scale is easy to use, having a high interobserver reliability that correlates with fasting insulin and body mass index (BMI) [23]. Many therapies have been attempted for AN, including topical and oral treatments. Topical retinoid (tazarotene) is considered first-line treatment; it is epidermopoietic and causes a reduction of the stratum corneum replacement time [19, 24]. Trichloroacetic acid (TCA) is a superficial chemical exfoliating agent causing destruction of the epidermis with subsequent repair and rejuvenation. TCA (15%) is caustic and causes coagulation of skin proteins leading to frosting. Precipitation of proteins leads to necrosis and destruction of epidermis, followed by inflammation and activation of wound repair mechanisms. This leads to reepithelialization with replacement of smoother skin [25]. Other topical treatments including calcipotriol, surgical excision, urea, salicylic acid, and triple-combination depigmenting cream (tretinoin 0.05%, hydroquinone 4%, and fluocinolone acetonide 0.01%) with sunscreens are other options [19].

Systemic therapies, oral retinoids (isotretinoin, acitretin), can be effective, probably through regulation of proliferation and differentiation of keratinocytes. Metformin and rosiglitazone are useful in AN characterized by IR; they reduce glucose production by increasing peripheral insulin responsiveness, reducing hyperinsulinemia, body weight, and fat mass and improving insulin sensitivity in peripheral muscles. Particularly, in this context metformin seems to function as a multipathway inhibitor of mechanistic target of rapamycin complex 1 (mTORC1) kinase affecting the pathogenesis of mTORC1-driven anabolic and hyperproliferative diseases of Western civilization (obesity, diabetes, etc.) [26]. A low-calorie diet, increasing physical activity and weight reduction, can improve the IR state, thus decreasing the severity of the skin disease [27].

### 4.2. Acne

Acne is a chronic inflammation of the folliculopilosebaceous unit (FPSU), due to hyperkeratosis and associated with sebaceous hypersecretion. It is more prevalent in adolescence and in female gender and is commonly located on face, shoulders, back, and chest with lesions that range from noninflammatory open or closed comedones (blackheads and whiteheads) to inflammatory lesions which may be papules, pustules, or nodules [28, 29]. Acne is the most common skin disease, being often widely and improperly considered to be a simple, self-limited disorder of adolescents [30]. However, acne may also be a common component of many systemic diseases or syndromes which are also usually linked to IR [31]. This is the case in seborrhea-acne-hirsutism-androgenetic alopecia (SAHA) syndrome, polycystic ovarian syndrome (PCOS), and hyperandrogenism, IR, and acanthosis nigricans (HAIR-AN) syndrome, conditions which may all require metabolic and hormonal evaluations as well as insulin-sensitizing medications [32]. In this context, PCOS represents the most common and well known clinical scenario which links IR and acne. Indeed, PCOS, which is typically characterized by hyperandrogenism, chronic anovulation, and polycystic ovaries, shows acne in 70% of cases, with 19% to 37% of women with moderate to severe acne meeting the criteria for this disorder [33, 34]. In particular, acne that originates or persists into adulthood and is refractory to conventional therapies should raise suspicion for underlying PCOS. Women with PCOS have abnormalities in the metabolism of androgens and estrogen and in the control of androgen production; moreover, PCOS is also associated with peripheral IR and hyperinsulinemia [35, 36]. Since insulin/IGF-1 receptors are expressed in epidermal keratinocytes, hyperinsulinemia may lead to an increased proliferation of basal keratinocytes within the FPSU duct inducing failure of terminal differentiation of follicular corneocytes, thus actively participating in acne pathogenesis. Furthermore, insulin also stimulates the synthesis of androgens, leading to high sebum production, a recognized correlate of acne severity [9, 37]. Moreover, IGF-1 is able to stimulate 5*α* reductase, adrenal and gonadal androgen synthesis, androgen receptor signal transduction, sebocyte proliferation, sebum production, and lipogenesis, affecting acne development [38, 39]. Indeed, IGF-1 is the growth promoter of puberty, playing a central role in acne and the induction of hyperandrogenism as highlighted by the fact that IGF-1-overtreated Laron patients usually exhibit hyperandrogenism [40]. Apart from PCOS, the close relationship between acne and IR is also highlighted by recent studies which showed that hyperglycaemic carbohydrates and insulinotropic milk/dairy products are linked to diabetes and may drive acne pathogenesis, promoting increased insulin/IGF-1 signaling and supporting also a connection between milk products, acne, and increased body mass index (BMI) [41–47]. Since high BMI is a major component of the metabolic syndrome, it is therefore not surprising that acne patients may often exhibit increased levels of serum glucose and insulin as well as IR, as recently reported by Del Prete et al. and Demir et al. [28, 48]. In this context, Western diet and lifestyle, two main actors of Western civilization, appear to be the linking points between acne, IR, and metabolic syndrome [49]. Indeed, acne is absent in populations consuming less insulinotropic palaeolithic diets that exclude grains, milk, and dairy products and exhibit much lower insulin/IGF-1 signalling [41, 50, 51]. Conversely, the Western diet is characterized by high glycaemic load and increased high levels of milk/dairy protein, containing abundant amounts of branched-chain amino acids (leucine, isoleucine, and valine). These two dietary stimuli are able to overstimulate a kinase termed mammalian target of rapamycin complex 1 (mTORC1). The activation of mTORC1 signalling is involved in both acne pathogenesis (altering sebaceous gland homeostasis with the promotion of cell growth and proliferation) and IR (stimulating the kinase S6K1, which negatively controls insulin signalling at the level of insulin receptor substrate-1 phosphorylation) [44, 49, 52]. Moreover, milk and dairy products act as enhancers of insulin/IGF-1 signalling, supporting sebaceous lipogenesis and acne aggravation through the derepression of the androgen receptor [45, 46, 53–55]. Indeed, a lipid-enriched sebaceous gland microenvironment may then promote excessive proliferation of* Propionibacterium acnes* and the lipophilic yeast* Malassezia furfur* with resultant inflammatory reactions of the pilosebaceous follicle [56]. Studies are also accumulating suggesting that low glycemic-load diet is able to improve acne [42, 57]. Moreover, there is evidence that a low glycaemic load diet can reduce the size of sebaceous glands, decrease inflammation, and diminish the expression of proinflammatory interleukin-8, all showing a positive influence on the clinical course and intensity of acne and sebum production [42, 58]. Overall, it has been interesting to note that the complex nutrient-regulated mTORC1 signalling pathway is the crucial molecular connection between acne, the Western diet, and IR. This is mediated through phosphoinositide 3-kinase (PI3-K), AKT kinase, the transcription factor FoxO1, androgen receptors, insulin, and IGF-1 [44]. A major role is played by FoxO1. It represses the androgen receptor, thus restricting access to that receptor. FoxO1 is inactivated by its extrusion from the nucleus to the cytoplasm, induced by high glycaemic load dairy protein consumption and increased insulin/IGF-1 signalling so that it is not able to suppress hepatic IGF-1 synthesis, inhibit the magnitude of androgen signalling, interact with regulatory proteins important for sebaceous lipogenesis, and regulate the activity of innate and adaptive immunity, as well as to act as a rheostat of mTORC1, the master regulator of cell growth, proliferation, and metabolic homoeostasis. All this drives increased protein and lipid synthesis, cell proliferation, cell differentiation including hyperproliferation of acroinfundibular keratinocytes, sebaceous gland hyperplasia, increased sebaceous lipogenesis, IR, and increased BMI, highlighting their parallel involvement in acne pathogenesis [59]. Interestingly, isotretinoin, one of the major acne treatments, is able to deeply influence mTORC1 pathway with its major effects linked to modifications of PI3K/AKT/FoxO1 signalling, further confirming their important role in acne development [60].

In conclusion, acne appears to develop in a metabolic environment with an increased activity of mTORC1 showing itself much more like a systemic rather than a skin disease. Therefore dermatologists may not solely focus on treating acne's skin pathology but should appreciate the great opportunity to introduce dietary and metabolic interventions so as to prevent more serious mTORC1-driven diseases of civilization like obesity, diabetes, and cancer.

### 4.3. Psoriasis

Psoriasis is a chronic skin inflammatory disease which is now considered a systemic immunomediated disorder. Patients suffering from psoriasis exhibit different clinical phenotypes that represent its dynamic spectrum [61]. The most common psoriasis type, accounting for up to 90% of cases, is psoriasis vulgaris, in which papulosquamous plaques are well delineated from surrounding normal skin. These plaques are salmon to pink lesions covered by white or silvery scales, which are usually distributed symmetrically on the extensor aspects of elbows and knees, scalp, and/or lumbosacral region [62]. Psoriasis patients are at high risk to develop cardiovascular and metabolic diseases including diabetes as well as metabolic syndrome [63]; conversely it is also well established that overweight and obesity are risk and exacerbating factors for psoriasis itself [64, 65]. However, the strict clinical connection between psoriasis and metabolic diseases (obesity, metabolic syndrome, etc.) is also underlined by analogies in their pathogenesis (chronic inflammation) showing factors like adipose tissue (AT) excess and IR as drive linking points. Indeed, AT is now recognized as a part of the innate immune system and adipocytokines, active factors secreted by AT, have an important role in the pathogenesis of both IR and psoriasis [63, 66, 67]. For example, adipocytokines such as leptin and adiponectin, which are able to regulate and affect insulin sensitivity through modulation of insulin signaling and the molecules involved in glucose and lipid metabolism, are deregulated in a very similar way in both psoriasis and obesity, highlighting the mechanisms of the possible common association with IR observed in those patients (e.g., plasma levels of adiponectin are decreased in obesity, psoriasis, IR, and type 2 diabetes) [68–71]. Moreover, these adipokines have also been found to regulate a huge variety of immune functions (cytokines production, T cells differentiation, etc.) showing an active role in the pathophysiology of psoriasis, highlighting the close connection of immunological and metabolic alterations, and linking the bases of psoriasis and IR [68, 72, 73]. Other adipocytokines apart from leptin and adiponectin may also be involved in the association between IR and psoriasis. This is the case with omentin, a protein produced by stromal vascular cells of visceral AT. It increases insulin sensitivity by stimulating insulin-mediated glucose uptake in human adipocytes. Indeed, serum levels of omentin inversely correlated with fat mass were found to be decreased in patients with psoriasis and negatively correlated with BMI and waist circumference [74]. Moreover, psoriasis patients also showed altered levels of further adipokines such as visfatin and resistin both of which have metabolic functions, also playing an important role in insulin sensitivity [75–77]. Another example of the tight relationship between psoriasis and IR is displayed by TNF-*α*, one of the major actors of psoriasis pathogenesis as demonstrated by the efficacy of anti-TNF-*α* treatments in psoriasis. TNF-*α* is also able to induce insulin signaling defects by acting on adipocytes and muscle cells, impair insulin signaling through inhibition of the tyrosine kinase activity of the insulin receptor, and suppress the secretion from adipocytes of adiponectin, an anti-inflammatory molecule that also functions in regulating insulin sensitivity [78, 79]. Furthermore, protein wingless-type MMTV integration site family member 5a (wnt5a) levels were shown to be upregulated in psoriatic skin lesions [80]. Wnt5a was also reported to be significantly higher in lean patients with psoriasis compared with lean healthy controls and in obese patients compared with obese healthy controls suggesting that, in psoriasis, an increase in wnt5a may contribute to the development of metabolic comorbidity [81]. Indeed, wnt5a is released from adipose tissue macrophages and was shown to be of importance in the development of IR [82]. Therefore it is not surprising that literature is accumulating that shows that patients with psoriasis (with or without psoriatic arthritis) commonly share obesity related complications such as metabolic syndrome, dyslipidemia, diabetes, and/or IR [67, 83, 84]. Particularly, Pereira et al. recently found a significant association between psoriasis and IR with an odds ratio of 2.63 of abnormal glucose homeostasis in psoriatics compared to controls, suggesting that treatments for psoriasis must also be designed to encourage lifestyle alterations such as diet modifications and exercise in addition to pharmacotherapy [85]. Moreover, insulin sensitivity indices were reported to be significantly lower in psoriatics, as compared with controls, with serum insulin level and IR indices demonstrating a significant positive correlation with the severity of psoriasis and being decreased after systemic treatments [86, 87]. These findings were recently confirmed by Gyldenløve et al. who showed that normal glucose-tolerant patients with moderate to severe psoriasis had significantly reduced insulin sensitivity compared with age-, gender-, and body mass index-matched healthy control subjects, supporting the notion that psoriasis per se may constitute a prediabetic condition [88]. Furthermore, the association between IR and psoriasis has been also reinforced by another recent study which showed that PCOS prevalence in a psoriatic cohort was higher than in nonpsoriatic women (47% versus 11%), highlighting that women with PCOS and psoriasis had a greater probability of IR, hyperinsulinaemia, and dyslipidaemia, as well as a more severe skin condition, than those with psoriasis alone [89]. IR has also been indicated as an important contributing mechanism to the development of psoriasis itself, driving not only cardiovascular comorbidities, but also its cutaneous phenotype. Particularly, Buerger et al. reported that IR directly contributed to the epidermal phenotype (hyperproliferation and altered differentiation of keratinocytes) seen in psoriasis, suggesting that key cytokines inducing IR in keratinocytes and kinases mediating their effects may represent attractive targets for novel antipsoriatic therapies [3]. Following this thinking, medications developed for diabetes had been studied in clinical trials for use in psoriasis therapy [90, 91]. In particular, thiazolidinediones, a novel class of insulin-sensitizing drugs, have demonstrated promise for treatment of psoriasis. Thiazolidinediones activate peroxisome proliferator-activated receptors (PPAR), a type of steroid/thyroid ligand-activated nuclear receptor that is expressed on human keratinocytes. In culture, ligands for peroxisome proliferator-activated receptor inhibit proliferation of both normal and psoriatic human keratinocytes [91] and newer thiazolidinediones, pioglitazone, and rosiglitazone have been demonstrated effective for treatment of psoriasis [92, 93] even if another recent study did not confirm these results [94].

However, the use of these PPAR activators in patients showing dermatologic diseases has to be deeply evaluated; for example, these drugs increase sebum production, which is not a favorable condition for acne patients [95].

In conclusion, psoriasis appears to be closely associated with IR. Psoriatic patients are at high risk of developing IR which is itself able to influence keratinocytes' homeostasis and psoriasis pathogenesis. There are numerous molecular factors responsible for this close connection with AT, and adipokines play a key role in both conditions.

## 5. Skin Diseases Potentially Associated with IR

### 5.1. Acrochorda

Acrochorda, or skin tags, are pedunculated soft brown papules most commonly seen on the neck and in the axillae and groin; they are frequently seen in association with acanthosis nigricans. Skin tags are harmless and do not usually cause pain, but they are unsightly and are a source of discomfort. A few studies have been reported regarding the abnormalities of carbohydrate and/or lipid metabolisms in patients with skin tags [96–98]. Indeed, Kahana et al. did not find an increased incidence with obesity but did report that those patients with acrochorda had greater impairment of carbohydrate metabolism [99]. Skin tags may be removed with cauterization, cryosurgery, ligation, or excision [100].

### 5.2. Androgenetic Alopecia

Androgenetic alopecia (AGA) is a hereditary thinning of hair induced by androgens in genetically susceptible individuals [101]. It has a polygenic pattern; the risk of AGA is known to be influenced by family history and genetic factors but precisely which gene(s) are involved is not clear [102]. In the presence of androgens, anagen phase is shortened, and hair follicles shrink or become miniaturized. With successive anagen cycles, the follicles become smaller, and short, nonpigmented vellus hairs replace thick, pigmented terminal hairs. The thinning may be diffuse, involving most of the scalp but being more marked in the frontal and parietal regions. In general, the frontal hairline is maintained with temporal recession in some women. Rarely, advanced thinning with the recession of frontal hairline occurs in virilization associated with markedly elevated circulating androgen levels [103]. Disagreements exist regarding the relationship between IR and AGA, although insulin was suggested to play a role in the regulation of cutaneous androgen metabolism and hair-growth cycle. In 2009, Nabaie et al. did not find an association between IR and AGA and suggested that IR may result from aging rather than AGA or due to the presence of metabolic syndrome [104]. Later, this was confirmed by other studies; no true association exists between AGA and IR, but their coexistence as in the case of metabolic syndrome could contribute to worsening of AGA [101]. On the other hand, Matilainen et al. reported a strikingly increased risk of hyperinsulinaemia and IR-associated disorders such as obesity, hypertension, and dyslipidemia in men with early onset of androgenetic alopecia (<35), compared with age-matched controls, supporting the hypothesis that early alopecia could be a clinical marker of IR [105]. Moreover, very recently Bakry et al. reported a significantly higher mean value of fasting serum insulin in AGA cases than in controls. Further 35% of cases and 19% of controls had IR with significant difference between both groups [106], confirming the results of previous studies which found a relationship between IR and early baldness [107–109]. Thus, a reduction in insulin sensitivity may play a pathogenetic role in the miniaturization of hair follicles, in the regulation of androgen metabolism and the hair growth cycle, all of which are relevant to the loss of scalp hair in male-pattern baldness, and [104, 109, 110] whether IR induces or promotes AGA needs to be clarified by further studies. However, it is advised that cases with early onset AGA should be assessed for components of metabolic syndrome and IR for early detection and control of cardiovascular risk factors [106].

### 5.3. Hidradenitis Suppurativa

Hidradenitis suppurativa (HS), also known as acne inversa, is a chronic follicular occlusive skin disorder characterized by recurrent abscesses, draining sinuses, and scarring tracts predominantly but not exclusively involving apocrine gland-bearing skin. HS mainly affects the intertriginous body areas including the axillae, the inguinal folds, the anogenital, the perineum, the inframammary regions, and the nape [111]. It is a common skin disease affecting 2%–4% of the population [112]. The etiology of HS is still poorly understood. However, it appears to be caused primarily by increased outer root sheath keratinocyte proliferation in the follicular portion of the FPSU leading to follicular duct occlusion. This is followed by rupture of the sebofollicular canal and extrusion of contents, including corneocytes, bacteria, yeast, sebum, and pilar residua ruptured hair follicles into the surrounding dermis, and the development of a polymorphous inflammatory infiltrate [113]. Increased prevalence of the metabolic syndrome is known in patients suffering from HS. Therefore, studies attempting to demonstrate primary hyperandrogenism as a cause of the disease have been complicated by the fact that the majority of these patients are obese. While this association further suggests obesity is an exacerbating factor [114], it is important to note that the foods of the Western diet that trigger the follicular occlusion and the IR are the same ones responsible for the obesity. The problem is not the obesity (thin patients also suffer from HS); it is the diet.

### 5.4. Hirsutism

Hirsutism, affecting up to 15% of women, is characterized by excessive growth of terminal hair in the androgen-sensitive skin regions. The presence of hirsutism in women can lead to significant psychological morbidity and can negatively influence the quality of life. The most common cause of hirsutism is PCOS, highlighting the close link and the importance in its pathogenesis played by IR [115]. However, idiopathic hirsutism (IH), the second most common cause of hirsutism, is defined as hirsutism associated with normal ovulatory function and normal circulating serum androgen concentrations [116]. Ünlühizarci et al. found a higher prevalence (18.7%) of impaired glucose tolerance among women with IH suggesting its association with IR [117]. These results were further confirmed by Abdel Fattah and Darwish who highlighted the presence of IR in IH as in PCOS, independent of a high BMI, suggesting that, despite not being the only responsible factor, IR can contribute to the aetiopathogenesis of IH [118].

## 6. Skin Diseases Anecdotally Linked to IR

### 6.1. Alopecia Areata

Alopecia areata (AA) is a common form of nonscarring alopecia involving the scalp and/or body, characterized by hair loss without any clinical inflammatory signs. In general population, the prevalence was estimated at 0.7–3.8% [119]. Alopecia areata has been described as being associated with diseases of the endocrine glands, various tension states and emotional shock, errors of refraction, vitiligo, and neurodermatitis and as a result of reflex irritations from focal lesions such as dental abscesses and from traumatic injuries [120]. Karadag et al., for the first time, showed that IR is significantly higher in AA than in controls. Increased inflammatory cytokines and hypothalamic-pituitary-adrenal axis activation may be responsible for this finding [121].

### 6.2. Vitiligo

Vitiligo, also called white spot disease or leukoderma, is a disease in which the skin loses its pigment due to the destruction of melanocytes. Vitiligo affects 1-2% of the world's population [122]. In 2011, one study evaluated the relationship between vitiligo and IR. A total of 96 subjects were included in the study, 57 patients with vitiligo and 39 subjects in an age- and a body mass index-matched control group. Comparison between the vitiligo and the control groups revealed that patients with vitiligo had higher IR (2.3 versus 2.0, *P* < 0.01), higher insulin and C-peptide levels (*P* < 0.001, *P* < 0.001, resp.), higher LDL/HDL ratio, and lower HDL-C levels (*P* < 0.01, *P* < 0.0001, resp.). The association between these two conditions is not yet clear [123].

## 7. Conclusions

Clinicians must always keep in mind that skin disorders may be a clue to internal alterations and/or diseases as is the case of acanthosis nigricans, alopecia, hirsutism, and so forth. On the other hand, numerous studies have also shown that some cutaneous diseases may be manifestations of systemic rather than simply skin disorders. Particularly, it is now well known that psoriasis, acne and hidradenitis suppurativa can be frequently associated with metabolic anomalies and/or comorbidities. In this review, we have shown the principal dermatologic conditions linked to IR. We wish to underline the necessity for the dermatologist to expand his attention beyond skin pathology so as to not miss the major opportunity for motivation of dietary and metabolic evaluations and interventions in order to properly support patients' health.

## Figures and Tables

**Table 1:** Skin diseases associated with insulin resistance.

Skin disorders and insulin resistance (IR)		
Conditions strongly associated with IR	Conditions potentially associated with IR	Conditions anecdotally linked to IR
Acanthosis nigricans	Acrochordons	Alopecia areata
Acne	Androgenetic alopecia	Vitiligo
Psoriasis	Hidradenitis suppurativa	
	Hirsutism	
	Hyperandrogenism	
